# Semi-automated histomorphometric quantification of acetabular labral surface roughness

**DOI:** 10.1007/s00418-026-02546-x

**Published:** 2026-11-13

**Authors:** Abdulaziz A. Alomiery, Sameerah Shaheen, Yousef A. Almajed, Ghazi Alanazi, Abduelmenem Alashkham

**Affiliations:** 1 Basic Sciences Department, Prince Sultan Bin Abdulaziz College for Emergency Medical Services, King Saud University, P.O. Box 25063, Riyadh, Saudi Arabia; 2 Department of Anatomy and Stem Cell Unit, College of Medicine and King, Saud University Medical City, King Khalid University Hospital, King Saud University, P.O. Box 2925, Riyadh, Saudi Arabia; 3 College of Science and Health Professions, King Saud Bin Abdulaziz University for Health Sciences, Riyadh, Kingdom of Saudi Arabia; 4 King Abdullah International Medical Research Center (KAIMRC), Riyadh, Kingdom of Saudi Arabia; 5 Edinburgh Medical School: Anatomy Department, College of Medicine and Veterinary Medicine, University of Edinburgh, Edinburgh, UK

**Keywords:** Acetabular labrum, Histomorphometry, Surface roughness, Histopathology, Digital image analysis, Fibrocartilage

## Abstract

Histopathologic surface changes, including fibrillation, fraying, undulation, and disruption, are important indicators of tissue wear and acetabular labral degeneration. Surface assessment, however, commonly relies on descriptive or semiquantitative grading and may be partly observer-dependent when applied to naturally curved fibrocartilaginous surfaces. This study developed and evaluated a semi-automated histomorphometric workflow for quantifying acetabular labral articular surface roughness using histologic images. Sixteen human hemipelves from nine embalmed cadavers were divided into eight anatomical segments and grouped into superior, anterior, inferior, and posterior quadrants. Sections were stained with haematoxylin and eosin and Safranin O/Fast Green, scanned at high resolution, and analysed using Fiji/ImageJ and Python. The labral articular surface was isolated, skeletonised into a one-pixel-wide line, and exported as coordinates. A specimen-specific LOWESS-smoothed reference contour was generated, and roughness percentage was calculated from the mean absolute pointwise deviation between the original and smoothed profiles. Roughness percentage did not differ significantly across anatomical quadrants. In contrast, it differed significantly across histopathologic surface severity levels, with severely degenerated surfaces showing higher values than normal, mild, and moderate surfaces. Intra- and inter-observer reliability was excellent, with intraclass correlation coefficients of 0.994 and 0.962, respectively. This workflow provides a reproducible quantitative extension to conventional histopathologic grading with potential future application to other curved, load-bearing fibrocartilaginous tissues.

## Introduction

The acetabular labrum is a fibrocartilaginous structure attached to the acetabular rim and contributes to hip joint stability, maintenance of the suction seal, load distribution, fluid pressurisation, and protection of the adjacent articular cartilage (Bsat et al. 2016; Henak et al. 2011; Storaci et al. 2020). Labral integrity is therefore important for normal hip biomechanics, whereas degeneration and tearing are commonly associated with hip pain, femoroacetabular impingement, dysplasia, chondral damage, and osteoarthritis (Bsat et al. 2016; Groh and Herrera 2009; Chahla et al. 2016). Histologically, the labrum is not a uniform structure; its collagen organisation, proteoglycan distribution, surface morphology, vascularity, innervation, and regional architecture vary in relation to mechanical function and anatomical position (Petersen et al. 2003; Alzaharani et al. 2014; Alomiery et al. 2025).

Histopathologic assessment remains important for characterising acetabular labral degeneration. Surface changes, including loss of smoothness, fraying, fibrillation, undulation, fissuring, and disruption, are among the principal indicators of tissue wear and degeneration (Alomiery et al. 2025; Pauli et al. 2011; Pritzker et al. 2006). However, these features are commonly assessed using descriptive or semiquantitative ordinal grading systems. Although such systems are biologically informative, they depend partly on observer judgement and may have limited sensitivity for detecting subtle differences in surface irregularity. Similar limitations have been recognised in other load-bearing fibrocartilaginous tissues, including the knee meniscus, where surface integrity is an important component of degeneration grading but is generally evaluated using semiquantitative categories (Pauli et al. 2011).

Quantitative assessment of the acetabular labral surface presents a particular methodologic challenge because the surface is naturally curved rather than flat. Its geometry may vary according to anatomical region, biomechanical loading, tissue size, section orientation, and the degree of degeneration. Consequently, comparison with a predefined flat or uniform reference surface may incorrectly attribute normal anatomical curvature to pathologic roughness. A suitable analytical method should therefore preserve the broad contour of each individual surface while isolating smaller-scale irregularities that may represent fibrillation, fraying, lamellar disruption, fissuring, or surface breakdown (Alomiery et al. 2025).

Quantitative surface texture and roughness methods have been applied to articular cartilage and meniscal degeneration, demonstrating that surface morphology can provide biologically meaningful information beyond conventional visual grading (Alomiery et al. 2025; Adler et al. 1992; Otani et al. 2022; Trudel et al. 2003; Ylitalo et al. 2019). These approaches have included histologic contour analysis, anatomically defined reference points, idealised surface contours, ultrasound-based assessment, and three-dimensional imaging. However, some require specialised imaging or assumptions about the expected surface geometry, and reproducible methods designed specifically for naturally curved histologic surfaces remain limited.

This study extends our previous histopathologic investigation of the human acetabular labrum by developing a semiautomated histomorphometric workflow to quantify articular surface roughness. The workflow uses a specimen-specific LOWESS-smoothed reference contour to retain the broad anatomical curvature of each surface while reducing local high-frequency irregularities. Each original surface profile is therefore compared with its own curvature-adjusted reference rather than with a predefined flat line. The aims of this study were: (1) to develop and evaluate a semiautomated histomorphometric workflow for quantifying acetabular labral articular surface roughness; (2) to determine whether roughness percentage differed across the superior, anterior, inferior, and posterior anatomical quadrants; and (3) to evaluate whether quantitative roughness percentage corresponded with histopathologic surface severity. We hypothesised that roughness percentage would not differ substantially across anatomical quadrants, consistent with the previously observed circumferential distribution of surface histopathology (Alomiery et al. 2025), but would increase with greater histopathologic surface severity.

## Materials and methods

### Specimen selection

Sixteen hemipelves (10 males, 6 females; mean age: 79.3 years; age range: 63–99 years) were obtained from nine embalmed cadavers provided by the Department of Anatomy, University of Edinburgh, in accordance with the Human Tissue (Scotland) Act 2006 guidelines (ethical approval code: ANATED_0033). Each hemipelvis was dissected to establish a clear visualisation of the acetabular labrum, joint capsule, and articular cartilage.

### Tissue processing, sectioning, staining, and slide scanning

**Fig. 1:**
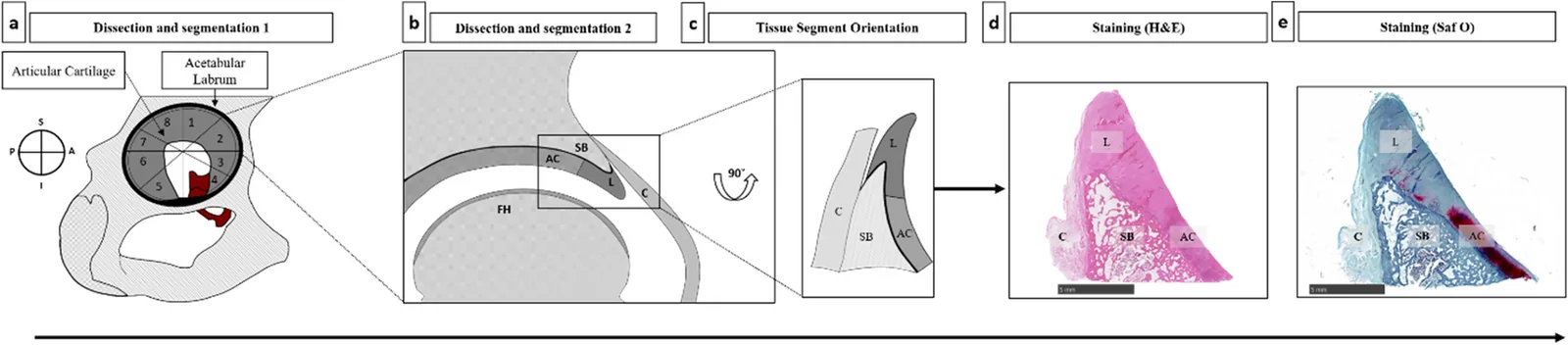
Acetabular segmentation, tissue orientation, and staining of labral specimens. **a** Schematic representation of the acetabulum divided into eight regional segments. **b** Schematic representation of the acetabular labrum and adjacent articular structures in relation to the femoral head. **c** Representative orientation of a tissue segment containing the labrum, capsule, articular cartilage, and subchondral bone. **d** Haematoxylin and eosin (H&E)-stained section showing the labrum, capsule, articular cartilage, and subchondral bone. **e** Safranin O/Fast Green-stained section used to support identification of the articular surface and labral-cartilage transition. Scale bars in panels (**d**) and (**e**): 5 mm. L, labrum; C, capsule; AC, articular cartilage; SB, subchondral bone; FH, femoral head

Each hemipelvis was divided into eight acetabular segments using a vertical cut from the posterior end of the transverse acetabular ligament, a horizontal cut at its midpoint, and two diagonal cuts through each quadrant. To ensure consistency between specimens, regional segments were grouped according to the anatomical regions they represent: segments 8 and 1 represent the superior region, segments 2 and 3 the anterior region, segments 4 and 5 the inferior region, and segments 6 and 7 the posterior region (Fig. 1).

Each of the eight regional segments was fixed in 10% neutral buffered formalin (pH 7.4; Sigma-Aldrich, Merck Life Science, Gillingham, UK) at 4 °C for 24 h. Decalcification was performed using Decalcifying Solution-Lite (Sigma-Aldrich, Merck Life Science, Gillingham, UK). Tissue segments were processed into paraffin wax blocks and stored at 4 ˚C. Each wax block contained a tissue segment with an intact labrum, capsule, cartilage, and bone. Sectioning was performed at 10 μm thickness using a Leica RM 2245 microtome (Leica Biosystems, Milton Keynes, UK) and high-performance microtome blades (Epredia™ MX35 Premier™; Fisher Scientific, Loughborough, UK). Haematoxylin and eosin (H&E) staining was used to evaluate overall tissue morphology, the histopathologic condition of the articular surface and collagen orientation (Fig. 1d). Safranin O/Fast Green staining was additionally used to support surface identification, as Safranin O preferentially stains glycosaminoglycan-rich cartilage matrix red, while Fast Green highlights bone and mineralised tissues in green to blue tones (Rosenberg 1971; Hyllested et al. 2002; Rutgers et al. 2010). In this study, Safranin O staining enabled clearer identification of the articular surface boundaries, facilitating consistent surface selection for roughness analysis (Fig. 1e). Slides were scanned at 40 × magnification using a NanoZoomer 2.0HT slide scanner (Hamamatsu Photonics K.K., Hamamatsu, Japan). The slides were then exported at high resolution using NDP.view2 software (Hamamatsu Photonics K.K., Hamamatsu, Japan).

Of the 128 potential regional samples, 115 histologic sections contained a sufficiently intact and clearly identifiable labral articular surface for quantitative analysis. Thirteen sections were excluded because the surface was incomplete, disrupted during processing, or could not be delineated reliably. Sectioning artefacts were identified by recognised features, including folds, compression, knife-induced tears or scores extending across the section, chatter parallel to the knife edge, and detached tissue fragments (Taqi et al. 2018). These were distinguished from histopathologic surface changes, including fibrillation, fraying, undulation, fissuring, or disruption, based on their morphology and continuity with the surrounding labral tissue.

### Histopathologic surface severity grading

The histopathologic surface grading used for comparison was derived from the Modified Pauli's Grading System (Pauli et al. 2011; Alomiery et al. 2025). Histologic grading was performed by two observers, with any scoring discrepancies resolved through discussion and consensus. Reliability was assessed using 13 randomly selected samples, with excellent intra- and inter-rater reliability for the total labral grade (ICC = 0.980 and 0.931, respectively) (Alomiery et al. 2025).

The surface component assessed the labral articular surface and lamellar layer using four ordinal levels: 0 = smooth surface; 1 = slight fibrillation or slightly undulating surface; 2 = moderate fibrillation or markedly undulating surface; 3 = severe fibrillation or disruption. The most severe surface change present in the assessed region was recorded. These four levels served as the categorical comparator for quantitative roughness percentage (Table 1).

**Table 1:** Histopathologic surface severity levels used for comparison with quantitative roughness percentage (Pauli et al. 2011; Alomiery et al. 2025)

Score	Surface histopathology category	Operational description
0	Smooth	Intact, smooth, non-frayed articular surface
1	Slight fibrillation or slightly undulating	Early surface irregularity without marked disruption
2	Moderate fibrillation or markedly undulating	More evident fibrillation, marked undulation, or surface irregularity
3	Severe fibrillation or disruption	Surface fissuring, severe fraying, disruption, or tears

### Image analysis: semi-automated roughness measurement workflow

The workflow was designed to quantify local surface irregularity while accounting for the naturally variable length and curvature of the acetabular labrum articular surface. The method was conducted in three stages: (1) selection of the labral articular surface, (2) isolation and extraction of a one-pixel surface line, and (3) Python-based feature analysis.

#### Selection of the labral articular surface

**Fig. 2:**
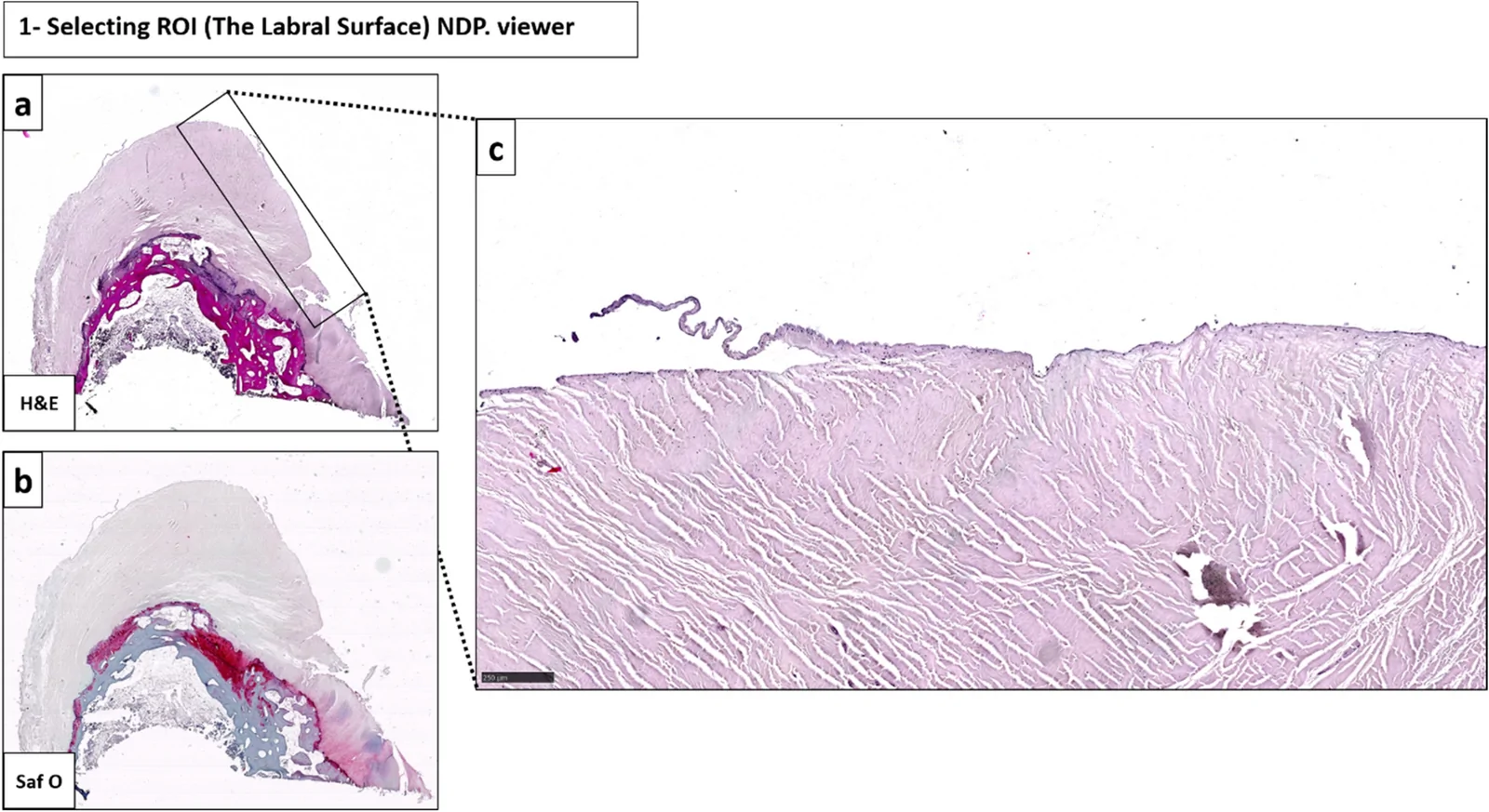
Selection of the labral articular surface. **a–b** Histologic sections of the acetabular labrum stained with **a** haematoxylin and eosin (H&E) and **b** Safranin O/Fast Green, illustrating selection of the labral articular surface using NDP.view2. The selected surface extended from the free edge of the labrum toward the labral-cartilage junction. **c** Higher-magnification H&E image showing the selected labral articular surface boundaries used for subsequent image processing. Scale bar in panel (**c**): 250 µm

This step aimed to define the starting and ending points of the labral articular surface for subsequent surface analysis. The analysis targeted the articular side of the acetabular labrum, extending from the free edge of the labrum to the labral-cartilage junction (Fig. 2a). The transition between the labrum and the adjacent articular cartilage has been described as fibrocartilaginous tissue containing isogenous groups of chondrocytes (Seldes et al. 2001). Therefore, the endpoint of the labral surface was defined as the point at which the tissue showed clear cartilage morphology without intervening labral fibres (Fig. 2a, b). Safranin O staining was used to better delineate cartilage structures because it preferentially stains proteoglycans and glycosaminoglycans (GAGs) red, thereby allowing clearer identification of the labral-cartilage transition (Fig. 2b) (Rosenberg 1971)**.**

#### Surface isolation and coordinate extraction in Fiji/ImageJ

**Fig. 3:**
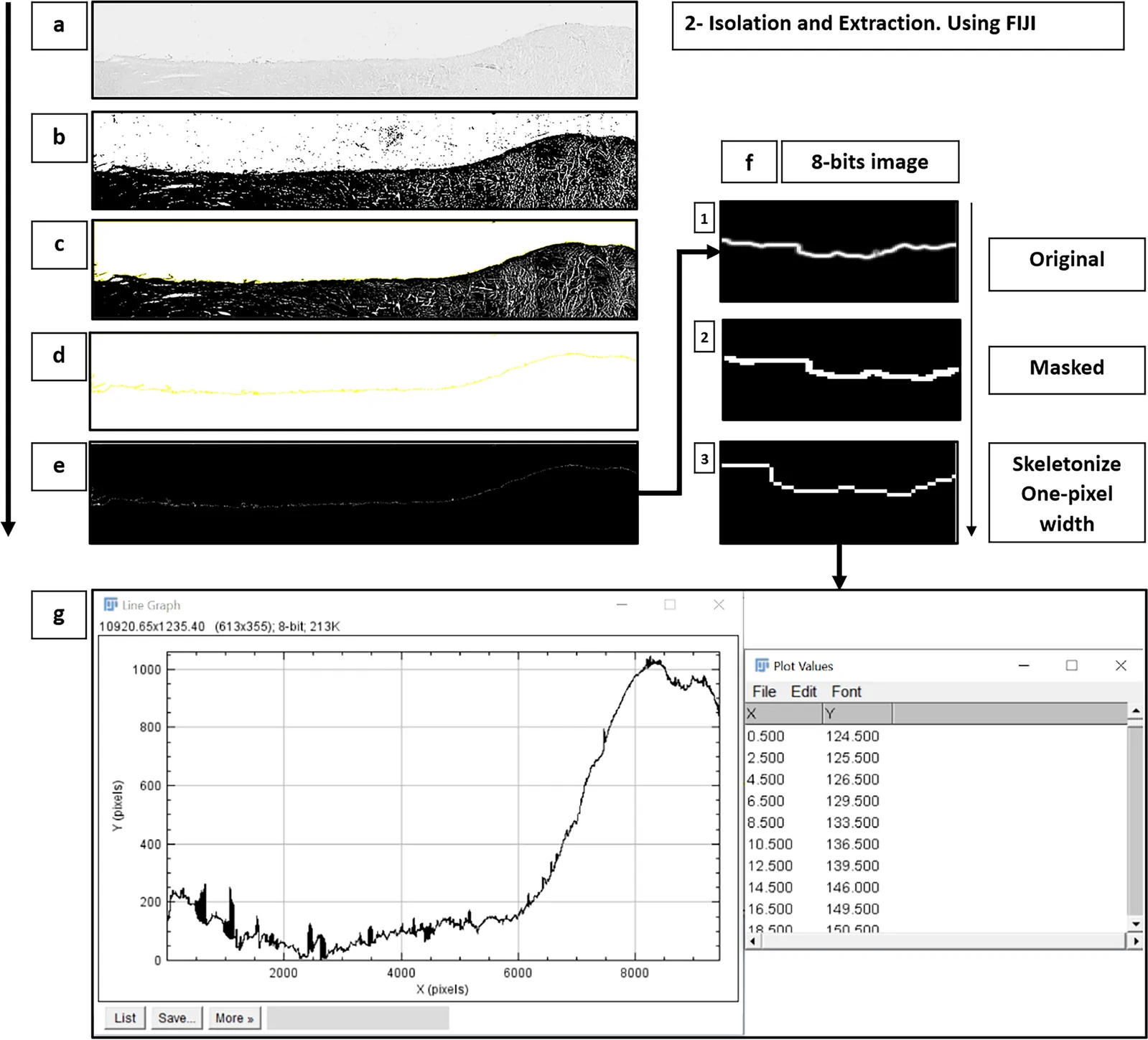
Surface isolation and coordinate extraction workflow in Fiji/ImageJ. **a** Imported histologic image of the selected labral articular surface following calibration to micrometres and conversion to 8-bit format. **b** Binary conversion of the selected surface using the Huang thresholding method. **c** Surface selection using the Wand tracing tool. **d, e** Clearing the selected surface region and surrounding background to remove external outliers and isolate the labral surface. **f1, f2** Binary mask generated from the processed surface to separate the selected labral surface from the background and enhance surface-feature visibility. **f3** Skeletonised surface profile, with the selected surface reduced to a one-pixel-wide line. **g** Line graph generated from the skeletonised surface profile using the Analyze Line Graph tool, from which the corresponding (x, y) coordinates were retrieved and exported for subsequent roughness analysis

This step aimed to isolate the labral surface and extract its corresponding (x, y) coordinates. First, the imported images were calibrated to micrometres in Fiji/ImageJ (National Institutes of Health, Bethesda, MD, USA) using the scale bar present on each image after export from NDP.view2. Second, using Fiji, the selected labral surface was cropped from the imported image, and the image was converted to 8-bit format (Fig. 3a). Third, a binary image was generated by thresholding. To improve reproducibility, the Huang thresholding method (Huang and Wang 1995) was used to obtain a binary representation of the selected surface (Fig. 3b). Fourth, the labral surface was selected using a Wand (tracing) tool (Fig. 3c). Fifth, the selected area and the area outside the selection were cleared to remove external outliers, and the remaining surface region was filled in black. As a result, the labral surface was delineated against a clear background (Fig. 3d, e).

Sixth, the image was converted into a binary mask to isolate the selected labral surface from the background and enhance surface-feature visibility. The masked image was then skeletonised, reducing the surface line to a one-pixel-wide structure to better represent its surface profile (Fig. 3f1–f3).

Seventh, using the Create Selection tool, the skeletonised surface line was overlaid onto the original image for visual inspection of its symmetry and alignment. Finally, using the Analyze Line Graph tool, the selected surface line was plotted, and its corresponding (x, y) coordinates were retrieved, collected for each labral segment, and tabulated in Microsoft Excel (Microsoft Corp., Redmond, WA, USA) for subsequent roughness analysis (Fig. 3g).

#### Python-based feature analysis

This step aimed to analyse the extracted (x, y) coordinates and define labral surface roughness by comparing the original surface profile with a specimen-specific smoothed reference contour. First, Python scripts were used to process the exported (x, y) coordinate data for each labral surface. The first notebook separated paired x- and y-coordinate columns into individual specimen-level worksheets and standardised the worksheet names. As image calibration was performed in Fiji/ImageJ before coordinate extraction, the exported coordinate values were already expressed in micrometres and were preserved during this data-organisation step.

Python-based analysis was conducted using Python version 3.14.6 in a Jupyter Notebook environment. Data organisation and tabulation were performed using pandas version 2.2.2, NumPy version 1.26.4, and openpyxl version 3.1.5. LOWESS smoothing was implemented using statsmodels version 0.14.6 through statsmodels.nonparametric.smoothers_lowess.lowess. The LOWESS-smoothed reference contour was interpolated onto the original x-coordinates using numpy.interp. The smoothing fraction was set to 0.13, and all other LOWESS parameters were left at their defaults.

**Fig. 4:**
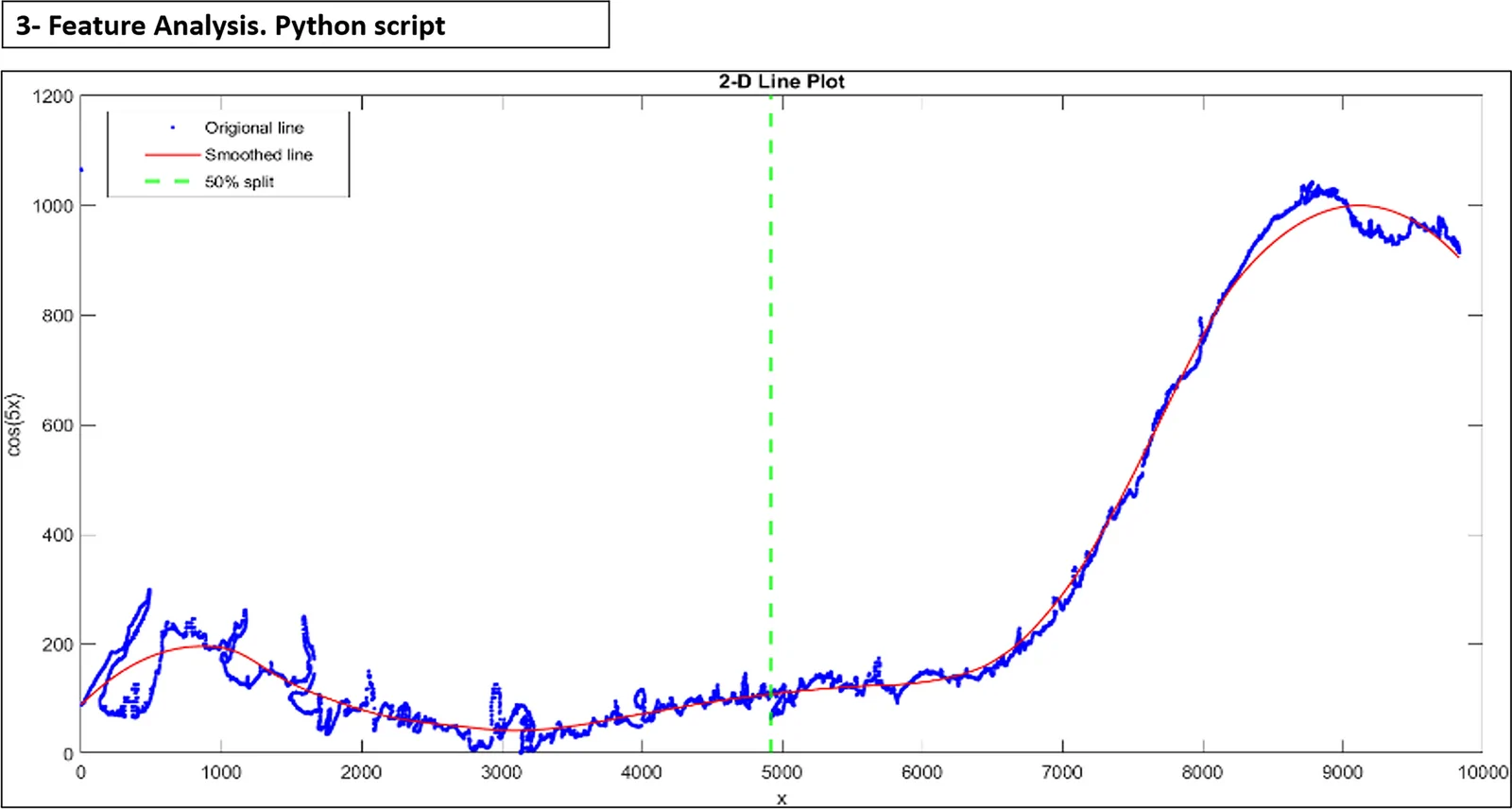
Python-based feature analysis of the extracted labral surface profile. Representative two-dimensional line plot generated from the extracted (x, y) coordinates of the labral articular surface. The original surface profile is shown in blue, while the LOWESS/LOESS-smoothed reference line is shown in red

The second notebook sorted the coordinates by x-position, generated a local regression smoothing curve using locally estimated scatterplot smoothing (LOWESS/LOESS), interpolated the smoothed curve onto the original x-coordinates, and calculated the absolute residual differences between the original surface line and the smoothed reference line. Because the original and smoothed lines occupied the same calibrated coordinate system, point-by-point comparison at corresponding x-positions was used to quantify local surface deviation. The mean absolute difference and standard deviation of the differences were then exported for each specimen. The LOWESS/LOESS smoothing fraction was fixed at 0.13 for all specimens. This value was selected following visual inspection of representative surface profiles because it provided an appropriate balance between preserving the broad anatomical contour of the labral surface and reducing local high-frequency irregularities (Fig. 4). The same smoothing fraction was applied to all specimens to maintain analytical consistency, while each LOWESS-smoothed line remained specimen-specific.

The LOWESS-smoothed line was treated as a specimen-specific reference that retained the large-scale curvature of the labral surface while reducing local high-frequency surface irregularities. This approach avoided assuming that all labral surfaces had the same degree of curvature. Instead, each surface was compared with its own curvature-adjusted reference, allowing the analysis to focus on local surface irregularity rather than differences in anatomical shape. The smoothed reference contour was interpolated onto the original x-coordinates using numpy.interp. The absolute residual difference between the original surface profile and the interpolated LOWESS-smoothed reference contour was calculated at each corresponding x-position. The mean of these absolute residuals was reported as the mean absolute deviation in micrometres:

$${\mathrm{mean}}\,\,\left| {{{y\_original - y\_smooth}}} \right|$$

Roughness percentage was additionally calculated as:

$$\begin{aligned}Roughness\,\% &= average{\left| {y\_original - y\_smooth} \right|} \mathord{\left/ {\vphantom {{\left| {y\_original - y\_smooth} \right|} {average\,y\,smooth \times 100}}} \right. \kern-\nulldelimiterspace} \\&\quad\;\; {average\,y\,smooth \times 100}\end{aligned}$$

where y original and y smooth represent the original extracted surface profile and the interpolated LOWESS-smoothed reference contour at corresponding x-positions, respectively. The mean y smooth value was defined as the average value of the LOWESS-smoothed reference contour across the analysed labral surface. This normalisation expressed the average local surface deviation relative to the average smoothed surface value. The original and smoothed profiles occupied the same calibrated coordinate system, and no vertical translation or baseline adjustment was applied before the pointwise comparison.

### Statistical analysis

#### Data

Measurement data were collected and tabulated in Microsoft Excel 2022 (Microsoft, Redmond, WA, USA). GraphPad Prism version 9.4.1 (681) (GraphPad Software Inc., San Diego, CA, USA) was used for graphical representation, and SPSS version 24 (IBM Corp., Armonk, NY, USA) was used for statistical analysis. Data were organised using Excel, GraphPad Prism, and Python-derived outputs.

Roughness percentage was compared across anatomical quadrants using one-way analysis of variance (ANOVA). Roughness percentage was also compared across the four histopathologic surface-severity levels using one-way ANOVA, followed by Tukey’s multiple-comparisons test where appropriate.

#### Reliability of roughness percentage measurements

**Fig. 5:**
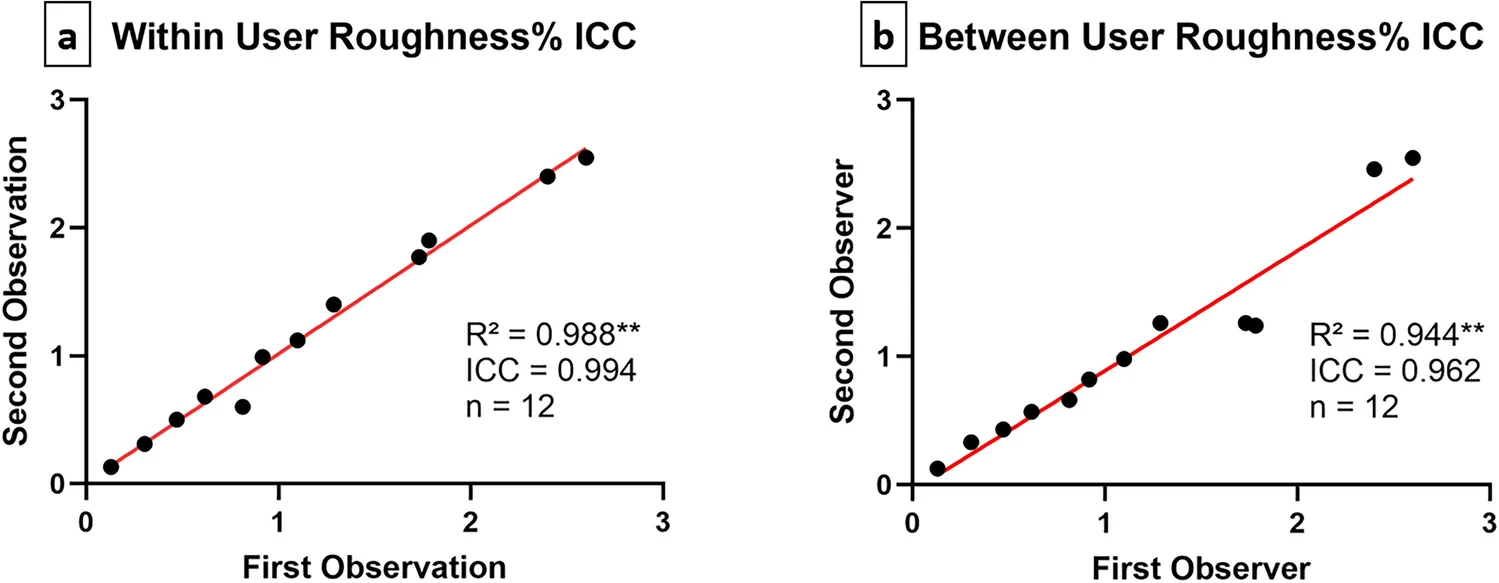
Reliability assessment of roughness percentage measurements. Reliability testing was performed on 12 selected labral surface samples to assess the reproducibility of the roughness percentage measurement. **a** Intra-rater reliability, comparing the first and second observations by the same observer, showed excellent agreement, with an ICC of 0.994 and R2 of 0.988. **b** Inter-rater reliability, comparing measurements between the first and second observers, also showed excellent agreement, with an ICC of 0.962 and R2 of 0.944. Each point represents one labral surface sample, and the red line represents the linear regression fit

Reliability testing was performed on 12 randomly selected samples, representing approximately 10% of the total sample, to assess within-observer and between-observer agreement for labral surface roughness measurements. For each reliability assessment, the selected labral surface samples were independently reprocessed through the full workflow, including surface selection, image processing, coordinate extraction, and roughness calculation. Within-observer reliability was excellent, with an intraclass correlation coefficient (ICC) of 0.994 and Cronbach’s alpha of 0.997. Between-observer reliability was also excellent, with an ICC of 0.962 and Cronbach’s alpha of 0.985. ICC values were calculated using a single-measure absolute-agreement model (Fig. 5).

## Results

The results are organised to address the main objectives of the study. First, roughness percentage was examined across anatomical quadrants to determine whether quantitative labral surface roughness was regionally concentrated or uniformly distributed around the acetabular circumference. Second, roughness percentage was compared across histopathologic surface severity levels to evaluate whether the semi-automated quantitative method corresponded with established histopathologic grading.

### Distribution of histopathologic surface severity

Among the 115 sections included in the quantitative analysis, 19 (16.5%) were classified as normal, 34 (29.6%) as mild, 11 (9.6%) as moderate, and 51 (44.3%) as severe. Descriptive examination of the grades within individual donors showed an uneven regional distribution of degeneration. Despite the advanced age of the donor cohort, degeneration was not uniformly distributed across regions within individual donors, with higher severity grades more frequently observed in the anterior and superior quadrants.

### Roughness percentage across anatomical quadrants

**Fig. 6:**
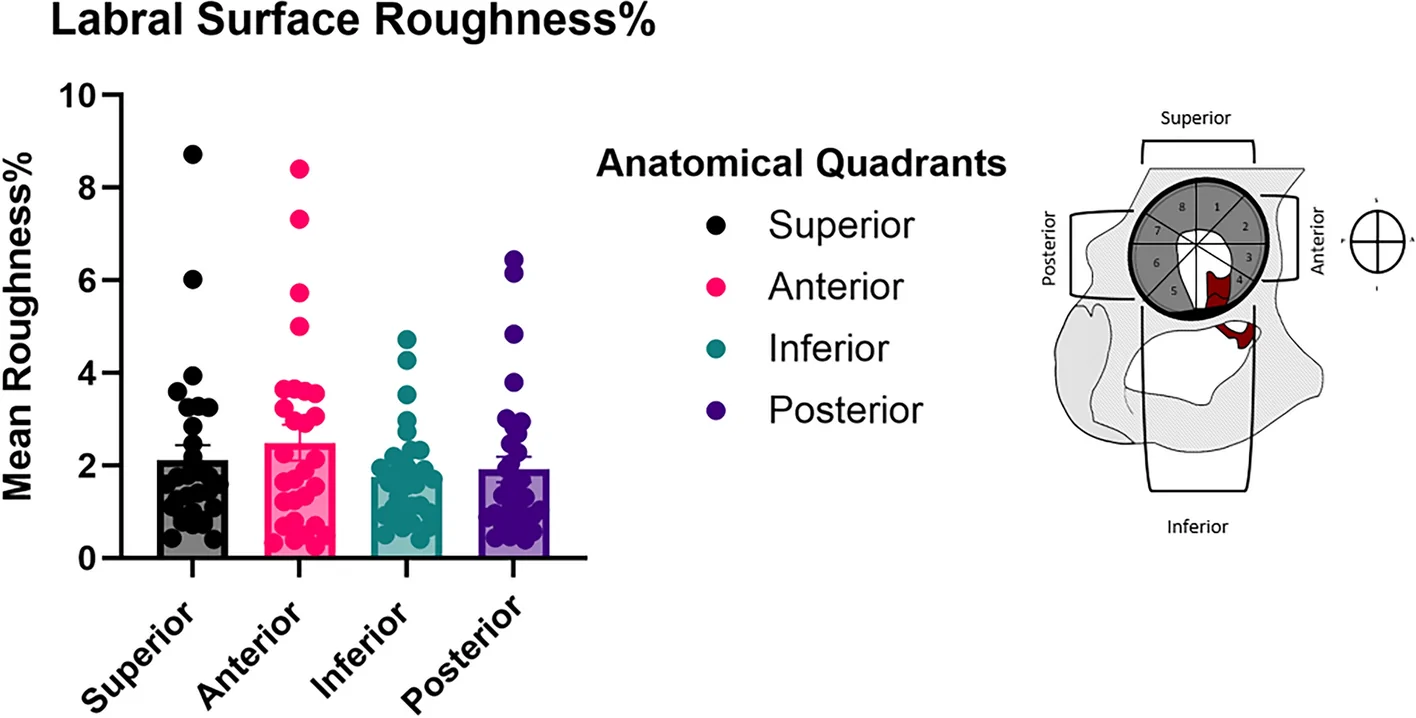
Roughness percentage across anatomical quadrants. Scatter bar plot showing labral surface roughness percentage across the superior, anterior, inferior, and posterior anatomical quadrants. One-way ANOVA showed no statistically significant difference in roughness percentage across anatomical quadrants, indicating that labral surface roughness was comparably distributed around the acetabular circumference. The schematic illustrates the anatomical segmentation corresponding to each quadrant

To determine whether labral surface roughness was concentrated within a specific anatomical region, roughness percentage was compared across the superior, anterior, inferior, and posterior labral quadrants. One-way ANOVA showed no statistically significant difference in roughness percentage across anatomical quadrants, *p* = 0.3404. Mean roughness percentage was 2.111 ± 1.763% in the superior quadrant, 2.490 ± 2.072% in the anterior quadrant, 1.750 ± 1.052% in the inferior quadrant, and 1.910 ± 1.555% in the posterior quadrant (Fig. 6 and Table 2**).** When roughness percentage was examined across anatomical quadrants within each histopathologic surface-severity group, no clear regional variation was apparent within any severity category (data not shown).

**Table 2:** Roughness percentage across anatomical quadrants

Anatomical quadrant	Mean ± SD roughness %
Superior	2.111 ± 1.763
Anterior	2.490 ± 2.072
Inferior	1.750 ± 1.052
Posterior	1.910 ± 1.555

One-way ANOVA showed no statistically significant difference in roughness percentage across the four anatomical quadrants (*p* = 0.3404)

### Roughness percentage across histopathologic surface severity levels

**Fig. 7:**
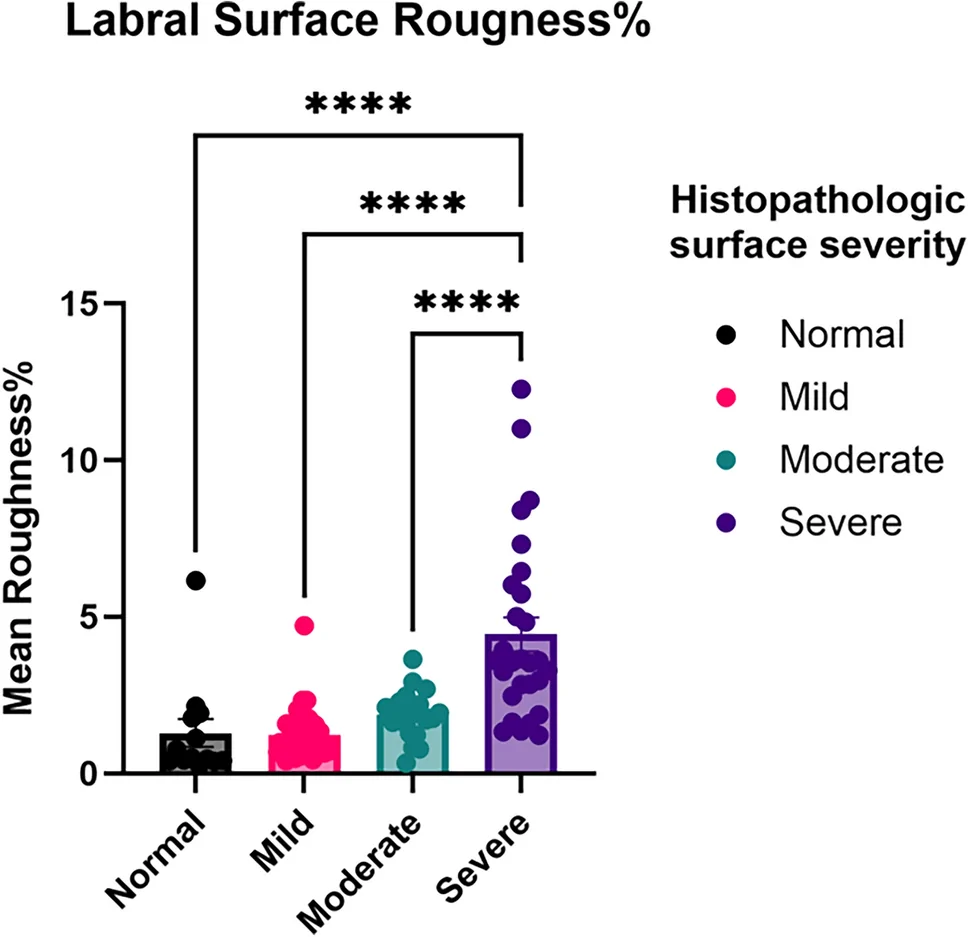
Roughness percentage across histopathologic surface severity levels. Scatter bar plot showing labral surface roughness across histopathologic surface severity levels. One-way ANOVA showed a statistically significant difference across severity levels, with severely degenerated surfaces exhibiting higher roughness percentages than normal, mild, and moderate surfaces

To evaluate whether the new quantitative roughness percentage method corresponded with histopathologic surface severity, roughness percentage was compared across normal, mild, moderate, and severe histopathologic surface severity groups. Mean roughness percentage was 1.282 ± 1.603% in normal surfaces, 1.222 ± 0.698% in mild surface changes, 1.867 ± 0.754% in moderate surface changes, and 4.439 ± 2.890% in severe surface changes (Fig. 7 and Table 3).

**Table 3:** Roughness percentage across histopathologic surface severity

Histopathologic surface severity	Mean ± SD roughness %
Normal	1.282 ± 1.603
Mild	1.222 ± 0.698
Moderate	1.867 ± 0.754
Severe	4.439 ± 2.890

One-way ANOVA showed an overall difference in roughness percentage across the four histopathologic surface-severity groups, F(3, 111) = 25.73, *p* < 0.0001. Tukey’s multiple-comparisons test showed that the severe group had significantly higher roughness percentages than the normal, mild, and moderate groups (all *p* < 0.0001). No significant differences were observed among the normal, mild, and moderate groups

One-way ANOVA showed a statistically significant difference in roughness percentage across histopathologic surface severity levels, F(3, 111) = 25.73, *p* < 0.0001, with severity level explaining 41.0% of the variance in roughness percentage (R2 = 0.4101). Tukey’s multiple comparisons test showed that the severe group had a significantly higher roughness percentage than the normal group, mean difference = 3.16%, *p* < 0.0001; the mild group, mean difference = 3.22%, *p* < 0.0001; and the moderate group, mean difference = 2.57%, *p* < 0.0001. No statistically significant differences were observed among the normal, mild, and moderate groups (Fig. 7 and Table 3).

These findings show that the roughness percentage method corresponds with histopathologic surface severity, particularly by distinguishing severely degenerated labral surfaces from lower-grade surface changes. The higher roughness observations in the normal and mild groups were not considered statistical outliers and were retained in the analysis. Their presence indicates that local quantitative surface irregularity may occur despite a lower overall histopathologic surface severity grade.

## Discussion

This study developed and applied a semi-automated histomorphometric workflow to quantify acetabular labral surface roughness, with normalisation to account for inherent variability in labral size and curvature. The principal findings were that the semi-automated workflow demonstrated excellent reliability and that surface roughness analysis distinguished increasing severity of labral surface degeneration according to the modified Pauli histopathologic grading system (Alomiery et al. 2025). In contrast, roughness percentage did not differ significantly across anatomical quadrants. Together, these findings support semi-automated surface roughness quantification as an objective extension of conventional histopathologic surface grading.

Although roughness percentage was higher in severely degenerated surfaces, it did not distinguish among normal, mild, and moderate surface-severity groups. This suggests that quantitative roughness and semiquantitative histopathologic grading capture related but distinct aspects of surface degeneration. The histopathologic surface score records the most severe local feature in a section; therefore, early focal fibrillation, slight undulation, or limited lamellar irregularity may increase the ordinal score without substantially increasing mean deviation across the entire surface profile. In contrast, the roughness metric quantifies the magnitude and spatial extent of deviation from the specimen-specific smoothed reference contour. It is therefore more responsive to extensive or pronounced surface disruption, such as severe fraying, fissuring, and tissue discontinuity. Accordingly, roughness percentage should be regarded as a complementary objective measure of advanced surface disruption rather than a replacement for semiquantitative grading of early or focal surface changes.

The current findings should be interpreted in relation to the known structure and function of the acetabular labrum. The acetabular labrum contributes to the hip fluid seal and load transmission, while its articular surface is subjected to repetitive compressive and shear forces (Petersen et al. 2003; Lertwanich et al. 2016; Alomiery et al. 2025). Degeneration of this surface may therefore reflect accumulated mechanical stress, aging, and/or joint pathology (Harris et al. 1979; Nepple et al. 2014; Philippon et al. 2005). Previous morpho-functional analysis demonstrated pronounced zonal differences, with greater degenerative changes observed in the inner zone adjacent to the articular surface (Alomiery et al. 2025). The significant association between the current quantitative roughness analysis and histopathologic surface severity provides an important validation of the proposed semi-automated workflow. In the present study, severely degenerated labral surfaces had a higher roughness percentage than mildly or moderately degenerated surfaces (Fig. 7 and Table 3). Thus, the method converts the visual impression of surface fibrillation, undulation, and disruption into a continuous quantitative metric that reflects the degree of surface degeneration.

The lack of significant quadrant-based differences suggests that surface roughness was not restricted to a single anatomical region of the acetabular labrum in this cadaveric cohort. This aligns with the global pattern observed in the previously published histopathologic analysis, where degenerative features were broadly distributed across the superior, anterior, inferior, and posterior quadrants rather than consistently concentrated in one region (Alomiery et al. 2025). This suggests that, at least in older cadaveric tissue, labral surface degeneration may represent a circumferential tissue response rather than a strictly regional phenomenon.

A key methodologic strength is that the method does not compare each surface to a predefined flat reference; instead, it accounts for inherent anatomical differences in surface curvature and size. In the present study, LOESS smoothing was employed to estimate the natural contour of the labral surface and generate a specimen-specific reference line. LOESS is a non-parametric regression method used to generate a smooth curve through scatterplot data (Cleveland 1981). Using this technique, the LOESS-smoothed line preserved the overall curvature of the labral surface while reducing local high-frequency irregularities, approximating a smoother reference contour for each specimen. By calculating the mean absolute pointwise deviation from this smoothed reference and expressing it relative to the average y-value of the LOESS-smoothed line, the method estimated roughness in relation to the overall contour of each selected labral surface segment. This makes the roughness percentage more geometrically interpretable and reduces the risk that differences in surface curvature and size influence the final normalised value.

Several important studies have advanced the quantitative assessment of articular surface degeneration by moving beyond descriptive grading systems. In articular cartilage, surface irregularity and roughness have been quantified using histology-based contour analysis, reference-point approaches, idealised surface contours, and three-dimensional imaging techniques, including contrast-enhanced microcomputed tomography (Hacker et al. 1997; Trudel et al. 2003; Ylitalo et al. 2019; Kauppinen et al. 2025). These studies provided an important methodologic foundation by showing that surface degeneration, including irregularity, fibrillation, and fissuring, can be translated into quantitative surface-based parameters rather than assessed only by visual grading.

Trudel et al. (2003) developed a histology-based method to quantify articular cartilage surface irregularity by manually delineating the cartilage contour, measuring distances from anatomically defined reference points, and calculating curvature-related changes along the surface. They then expressed surface irregularity as the percentage of contour segments exceeding a defined threshold. Ylitalo et al. (2019) used contrast-enhanced microcomputed tomography to quantify the three-dimensional microtopography of degenerated articular cartilage. Their method generated a reference surface and quantified the spatial relationship between this reference and the actual cartilage surface using parameters such as maximum void depth, integral void depth, root mean square roughness, and complex roughness. More recently, Kauppinen et al. (2025) developed the Cartilage Roughness Score, a microcomputed tomography-based method for assessing whole-surface articular cartilage damage relative to an estimated healthy surface.

However, many of these approaches were developed primarily for articular cartilage and often require specialised imaging, three-dimensional reconstruction, or tissue-specific assumptions about the expected surface contour. In contrast, a strength of the present study is that roughness quantification was performed using standard histologic sections, without microcomputed tomography or additional three-dimensional imaging. This makes the workflow more accessible and potentially adaptable to different histologic datasets and tissue types.

This is particularly relevant for the acetabular labrum, which is a soft fibrocartilaginous structure with a naturally variable surface contour rather than a relatively uniform articular cartilage surface. A normal labral surface is therefore not necessarily flat; it may be curved or undulating because of anatomical shape, local biomechanical loading, and sectioning orientation. This creates a specific methodologic challenge: the reference surface used for roughness analysis must distinguish true pathologic surface disruption from normal anatomical curvature.

Quantitative surface assessment has also been explored in another fibrocartilaginous tissue, the knee meniscus. Otani et al. (2022) measured meniscal surface roughness and showed that surface roughness correlated with Pauli’s histologic degeneration score and was associated with degeneration-related gene expression and biomechanical properties. This supports the broader concept that surface texture in fibrocartilaginous tissues can reflect biologically meaningful degeneration. Nevertheless, to our knowledge, quantitative roughness analysis of the acetabular labral articular surface has not previously been established.

The present study is therefore novel in adapting surface roughness quantification to the unique geometry of the acetabular labrum using a histology-based workflow and a specimen-specific LOESS-smoothed reference line. This allowed us to quantify roughness relative to the natural contour of each selected labral surface segment, rather than to a predefined flat reference.

This approach may also be adaptable to other load-bearing tissues with curved or irregular surfaces, including the glenoid labrum, knee meniscus, tendon enthesis, articular cartilage, and other fibrocartilaginous structures. A tissue-specific smoothed reference-line approach could support cross-tissue comparisons and quantitative evaluation of degeneration or repair.

While the novel method presented in this study demonstrated reliable surface roughness measurement, several limitations should be acknowledged. First, the workflow remains semi- rather than fully automated. Selection of the labral surface, thresholding decisions, and verification of the extracted one-pixel surface line still require observer input. Second, LOESS smoothing requires selection of a smoothing parameter, and future work could optimise this parameter systematically rather than relying primarily on visual verification. Third, the samples were obtained from embalmed cadavers of older age, which may limit direct generalisation to younger clinical populations.

Despite these limitations, the present study supports the potential of surface roughness percentage as a quantitative metric for assessing degeneration across anatomically variable soft tissues. Future work could focus on automated segmentation, systematic optimisation of thresholding and smoothing parameters, and correlation with clinical, biomechanical, histologic, and radiologic markers of degeneration.

## Conclusions

This study presents a semi-automated histomorphometric method for quantifying acetabular labral surface roughness. Roughness percentage was similarly distributed across anatomical quadrants but was significantly higher in labral surfaces with severe histopathologic fibrillation or disruption. By combining calibrated digital histology, Fiji/ImageJ surface extraction, LOESS smoothing, and Python-based pointwise deviation analysis, the method provides a continuous and reproducible measure of labral surface irregularity. The key technical contribution of this workflow is not simply measuring surface irregularity but also quantifying surface micro-irregularities on a naturally curved, non-flat histologic surface. This supports its potential as a transferable quantitative tool for analysing curved and load-bearing soft tissue surfaces.

## Data Availability

No datasets were generated or analysed during the current study.

## Code availability

The Python scripts and Jupyter notebooks used for coordinate organisation, LOWESS smoothing, interpolation, pointwise deviation analysis, and roughness-percentage calculation are available from the corresponding author on reasonable request.
